# Histopathological, molecular, clinical and radiological characterization of rosette-forming glioneuronal tumor in the central nervous system

**DOI:** 10.18632/oncotarget.22646

**Published:** 2017-11-24

**Authors:** Chenlong Yang, Jingyi Fang, Guang Li, Shaowu Li, Tingting Ha, Jiangfei Wang, Bao Yang, Jun Yang, Yulun Xu

**Affiliations:** ^1^ Department of Orthopedics, Peking University Third Hospital, Haidian District, Beijing 100191, China; ^2^ Department of Neurosurgery, Beijing Tiantan Hospital, Capital Medical University, Dongcheng District, Beijing 100050, China; ^3^ China National Clinical Research Center for Neurological Diseases (NCRC-ND), Dongcheng District, Beijing 100050, China; ^4^ Department of Neuro-pathology, Beijing Neurosurgical Institute, Capital Medical University, Dongcheng District, Beijing 100050, China; ^5^ Department of Pathology, Beijing Tiantan Hospital, Capital Medical University, Dongcheng District, Beijing 100050, China; ^6^ Department of Neuroradiology, Beijing Neurosurgical Institute, Capital Medical University, Dongcheng District, Beijing 100050, China; ^7^ Department of Radiology, Peking University Shougang Hospital, Shijingshan District, Beijing 100144, China

**Keywords:** rosette-forming glioneuronal tumor, brain tumor, central nervous system, spinal cord tumor, treatment

## Abstract

**Objective:**

A rosette-forming glioneuronal tumor (RGNT) is a rare entity originally described in the fourth ventricle. Recently, RGNTs occurring in extraventricular sites and those with malignant behaviors have been reported. The purpose of this study was to analyze the clinicoradiological and histopathological features, therapeutic strategies, and outcomes of RGNTs.

**Methods:**

We enrolled 38 patients diagnosed with RGNTs pathologically between August 2009 and June 2016. CT and MRI, including diffusion-weighted imaging and spectroscopy, were performed. The surgical treatment and histopathological and molecular features were assessed. Additionally, we searched the relevant literatures and performed a pooled analysis of individual patient data. The potential risk factors of prognosis were analyzed.

**Results:**

Our case series included 22 male and 16 female patients, with a mean age of 25.9 years. RGNTs involved the fourth ventricle (26.3%), cerebella (34.2%), supratentorial ventricular system (13.2%), spinal cord (10.5%), temporal lobe (10.5%), thalamus (7.9%), brain stem (7.9%), frontal lobe (5.3%), pineal region (5.3%), suprasellar region (2.6%), and basal ganglia (2.6%). Statistical analyses showed that pediatric age, purely solid appearance of the tumor, and inadequate resection (only partial removal or biopsy) were risk factors associated with progression events. Patients with subtotal resection appeared to do as well as those with gross total resection.

**Conclusions:**

RGNTs can occur nearly anywhere in the CNS, at both supratentorial and infratentorial sites. Maximal safe surgical resection should be emphasized for treatment; whilst aggressive resection with the goal of complete resection may be unnecessary.

## INTRODUCTION

Mixed glioneuronal tumors of the CNS are rare low-grade tumors that consist of glial and neuronal cells at varying stages of differentiation [1]. Rosette-forming glioneuronal tumors (RGNTs) have been recently identified as an unusual variant of mixed neuronal-glial tumors, and they were first categorized as a novel tumor entity “*rosette-forming glioneuronal tumors of the fourth ventricle*” in the 2007 WHO classification of CNS tumors [2]. This nomenclature was based on the fact that RGNTs were originally described as occurring exclusively in the fourth ventricle, with limited extension into surrounding structures, including the cerebellar vermis, midbrain, and cerebral aqueduct [3–6]. However, the view has been challenged by the increasing number of subsequent case reports that have indicated the presence of this entity in various anatomical locations ranging from the cerebellar hemisphere and/or vermis to the pineal region, chiasma, lateral and third ventricle, hypothalamus, and spinal cord [4, 7–10]. Thus, in the 2016 edition of the WHO classification system, these tumors have been renamed to “*rosette-forming glioneuronal tumors*” histologically classified as grade I [11].

Despite the benign histological grade, little is known about the true nature of this newly recognized tumor entity. A few case reports have described malignant behaviors, such as tumor recurrence and dissemination [4, 12–14].

In the literature, only approximately 150 cases of RGNTs have been described, and these were limited to single-case reports or small case series. The clinical, radiological, and immunohistochemical features of RGNTs are yet to be well elucidated, and current treatment approaches and prognosis are still elusive owing to the paucity of studies. The purpose of this large-sample, single-center study was to increase the current knowledge about RGNTs.

## RESULTS

### Demographic and clinical characteristics

Our case series included 22 male and 16 female patients, with a mean age of 25.9 years (SD = 15.6; range, 2–64 years). The mean symptom duration was 21.6 ± 39.2 months (range, 2 weeks-16 years). Clinical symptoms were localization-related, and headache was the most common. The demographic and clinical characteristics of the patients enrolled in the current study and patients reported in the literature are described in Table 1.

**Table 1:** Clinical characteristics of the RGNTs

Characteristics	Current study		Literature review		Total	
	n=38		n=153		n=191	
**Sex**	n	%	n	%	n	%
Male	22/38	57.9%	71/148	48%	93/186	50%
Female	16/38	42.1%	77/148	52%	93/186	50%
**Age (years)**	n	%	n	%	n	%
Mean (range; SD)	25.92 (2-64; 15.64)		28.61 (4-81; 15.67)		28.06 (2-81; 15.65)	
Pediatric (<18 years)	14/38	36.8%	38/148	25.7%	52/186	28.0%
Adult (≥18 years)	24/38	63.2%	110/148	74.3%	134/186	72.0%
Patients <26 years	23/38	60.5%	74/148	50%	97/186	52.2%
Patients ≥26 years	15/38	39.5%	74/148	50%	89/186	47.8%
**Clinical symptoms**	n	%	n	%	n	%
Asymptomatic	1/38	2.6%	11/117	9.4%	12/155	7.7%
Headache	19/38	50.0%	75/117	64.1%	94/155	60.6%
Ataxia	4/38	10.5%	38/117	32.5%	42/155	27.1%
Nausea/vomiting	8/38	21.1%	37/117	31.6%	45/155	29.0%
Vertigo	11/38	28.9%	21/117	17.9%	32/155	20.6%
Cranial nerve impairment	2/38	5.3%	20/117	17.1%	22/155	14.2%
Papilledema	1/38	2.6%	14/117	12.0%	15/155	9.7%
Visual disturbance	3/38	7.9%	14/117	12.0%	17/155	11.0%
Epileptic seizure	6/38	15.8%	8/117	6.8%	14/155	9.0%
Local pain	1/38	2.6%	6/117	5.1%	7/155	4.5%
Consciousness disturbance	0/38	0%	7/117	6.0%	7/155	4.5%
Extremity motor defect	2/38	5.3%	7/117	6.0%	9/155	5.8%
Extremity sensory defect	3/38	7.9%	5/117	4.3%	8/155	5.2%
Bladder dysfunction	0/38	0%	3/117	2.6%	3/155	1.9%
Anisocoria	0/38	0%	2/117	1.7%	2/155	1.3%
Precocious puberty	0/38	0%	1/117	0.9%	1/155	0.6%
Nystagmus	0/38	0%	1/117	0.9%	1/155	0.6%
Memory disorder	0/38	0%	1/117	0.9%	1/155	0.6%
Neck rigidity	0/38	0%	1/117	0.9%	1/155	0.6%
Fever	0/38	0%	1/117	0.9%	1/155	0.6%
Not mentioned	0/38	0%	36/153	23.5%	36/191	18.8%
**Duration**	n=37		n=76		n=113	
Range	2 weeks-16 years		1 day-20 years		1 day-20 years	
Mean ± SD (months)	21.6 ± 39.2		26.9 ± 51.8		25.2 ± 47.9	

### Radiological manifestations

**Figure 1:**
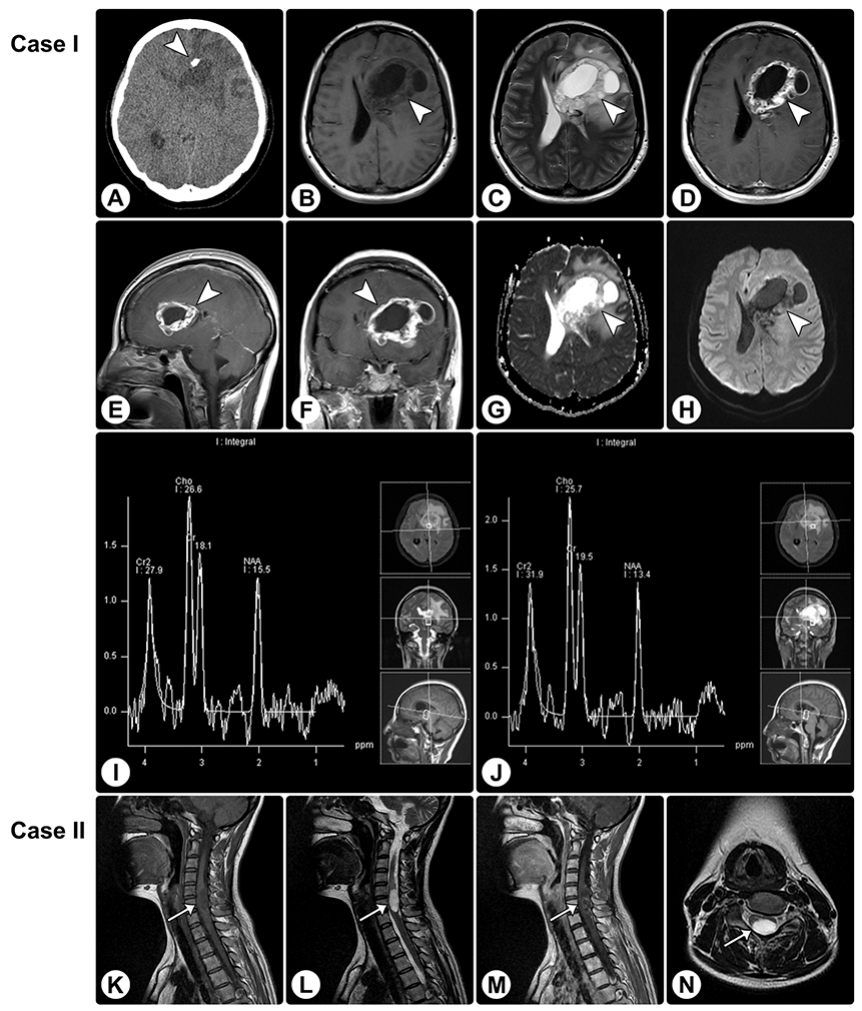
Rosette-forming glioneuronal tumor in the frontal lobe involving the lateral ventricle and rosette-forming glioneuronal tumor in the spinal cord **(A)** CT reveals a hypodense lesion (arrowhead) in the right frontal lobe involving the lateral ventricle, and focal calcification is visible. **(B and C)** MRI shows a cystic-sold lesion (arrowheads) with hypointensity on axial T1WI (B) and hyperintensity on axial T2WI (C). **(D–F)** The axial (D), sagittal (E), and coronal (F) contrast T1WI show heterogeneously remarkable enhancement in the solid portion of the tumor. **(G and H)** The apparent diffusion coefficient (ADC) map (G) and DWI (H) show facilitated diffusion. **(I and J)** MRS demonstrates an elevated choline value, reduced NAA value, and absence of lactate or lipid peaks. **(K–M)** MRI of another patient reveals an intramedullary mass (arrows) in the spinal cord, with hypointensity on sagittal T1WI (K), hyperintensity on sagittal (L) and axial **(N)** T2WI, and heterogeneous enhancement on sagittal contrasted T1WI (M).

**Figure 2:**
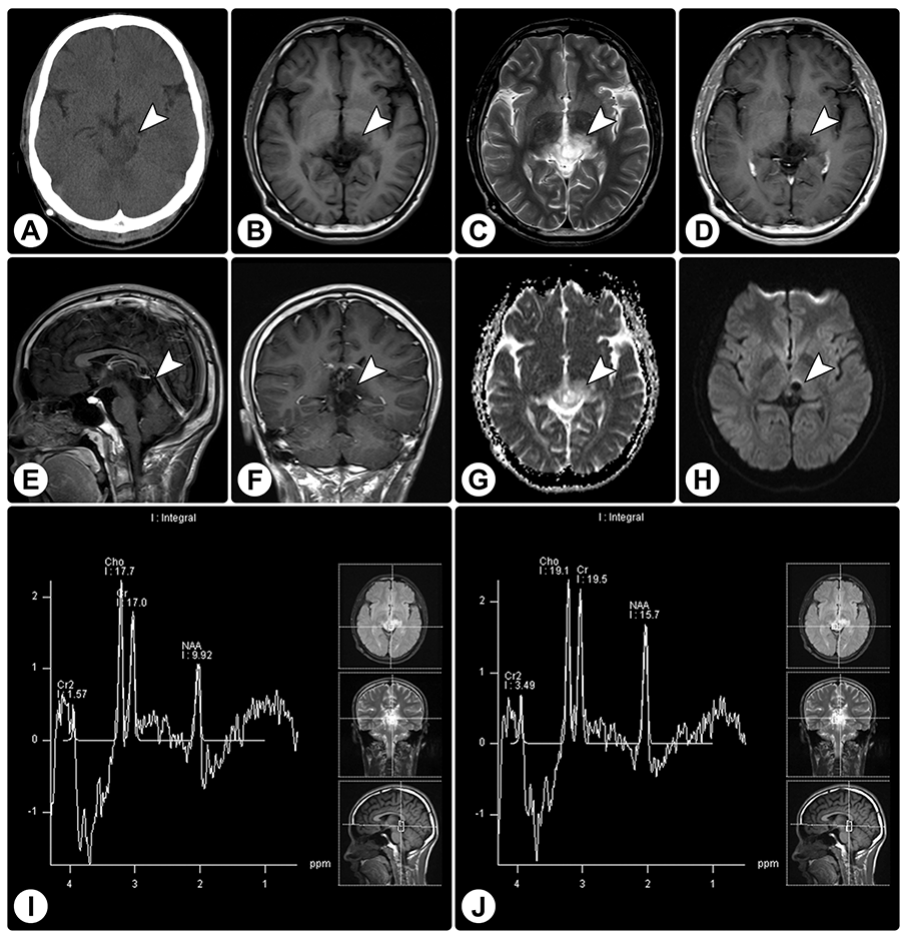
Rosette-forming glioneuronal tumor in the pineal region involving the tectum **(A)** CT reveals a slight hypodense lesion (arrowhead) in the pineal region involving the tectum, without calcification. **(B and C)** MRI shows a cystic-sold lesion (arrowheads) with hypointensity on axial T1WI (B) and hyperintensity on axial T2WI (C). **(D–F)** Axial (D), sagittal (E), and coronal (F) contrast T1WI show no significant enhancement. **(G and H)** The ADC map (G) and DWI (H) show facilitated diffusion. **(I and J)** MRS demonstrates an elevated choline value, reduced NAA value, and absence of lactate or lipid peaks.

**Figure 3:**
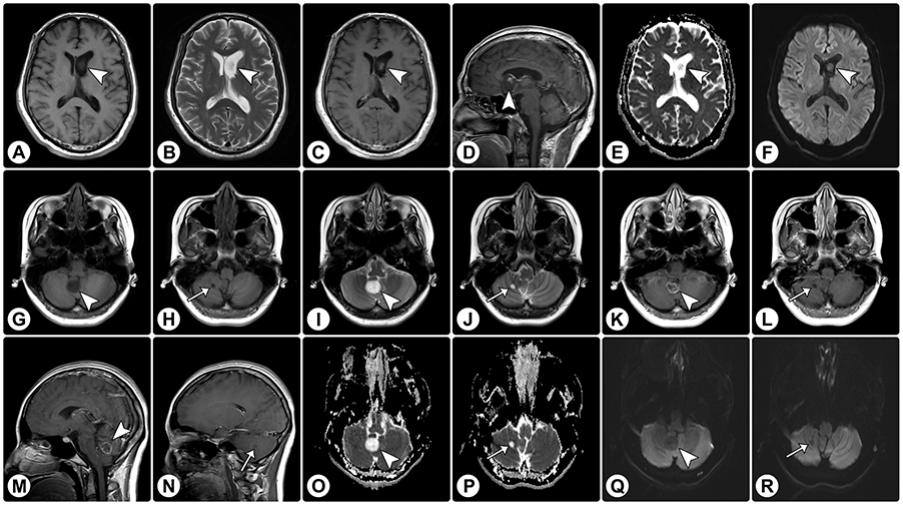
Rosette-forming glioneuronal tumor in the lateral ventricle and cerebellar rosette-forming glioneuronal tumor with a satellite lesion **(A-F)** MRI demonstrates a solid mass (arrowheads) in the lateral ventricle, with hypointensity on axial T1WI (A) and hyperintensity on axial T2WI (B). (C and D) Axial (C) and sagittal (D) contrast T1WI show focal enhancement. **(**E and F) The ADC map (E) and DWI (F) show facilitated diffusion. **(G–J)** MRI of another patient reveals a solid mass in the cerebellar vermis (arrowheads) and a satellite lesion in the cerebellar hemisphere (arrows); both of these show hypointensity on axial T1WI (G and H) and hyperintensity on axial T2WI (I and J). **(K–N)** On axial (K and L) and sagittal (M and N) contrast T1WI, the vermis lesion exhibits rim enhancement (arrowheads) and the satellite lesion shows no enhancement (arrows). **(O–R)** The ADC map (O and P) and DWI (Q and R) show facilitated diffusion.

**Figure 4:**
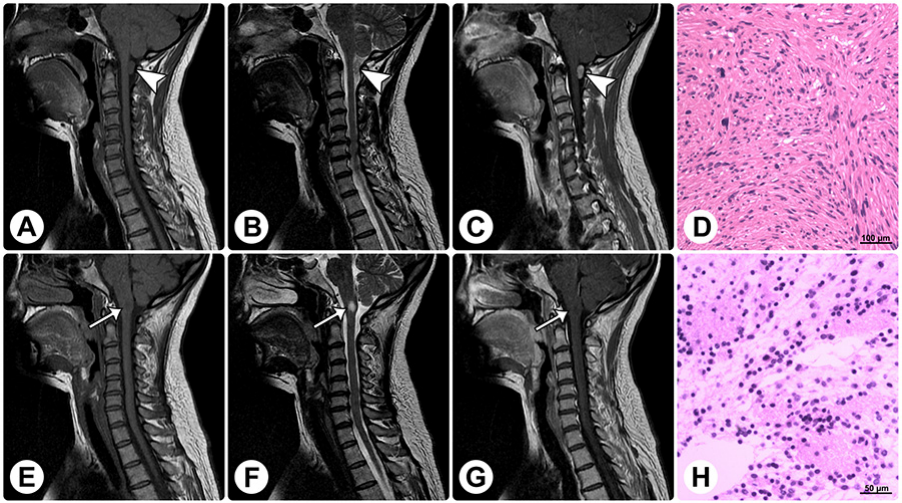
Radiological and histopathological profiles of a patient with concomitant rosette-forming glioneuronal tumor and schwannoma MRI reveals a spinal extramedullary solid mass at the C1 level (arrowheads) and an intramedullary solid mass in the medulla oblongata (arrows). **(A–C)** The former mass shows isointensity on sagittal T1WI (A), slight hyperintensity on sagittal T2WI (B), and homogeneously remarkable enhancement on sagittal contrast T1WI (C). **(E–G)** The latter mass shows isointensity on sagittal T1WI (E), remarkable hyperintensity on sagittal T2WI (F), and no enhancement on sagittal contrast T1WI (G). **(D and H)** Histopathological examinations of these 2 lesions are consistent with schwannoma (D) and RGNT (H), respectively. (Original magnification: D: 100×; H: 200×).

The radiological features of RGNTs are summarized in Table 2. RGNTs could be found nearly throughout the CNS. Cerebella (34.2%) and the fourth ventricle (26.3%) were most commonly involved, followed by supratentorial ventricular system (13.2%), spinal cord (10.5%), temporal lobe (10.5%), thalamus (7.9%), brain stem (7.9%), frontal lobe (5.3%), pineal region (5.3%), suprasellar region (2.6%), and basal ganglia (2.6%). Hydrocephalus was present in 36.8% of the patients. A small nodular satellite lesion in the cerebellar hemisphere was noted in one patient. On MRI, RGNTs showed the following three patterns: cystic pattern, solid pattern, and mixed cystic-solid pattern. The overwhelming majority of RGNTs showed hypointensity (94.7%) on T1-weighted imaging (T1WI) and hyperintensity (86.8%) on T2-weighted imaging (T2WI). After the administration of contrast medium, approximately a quarter of RGNTs demonstrated no enhancement, and the others showed heterogeneous (44.7%), rim (23.7%), or focal (7.9%) enhancement, which was associated with the cystic/solid nature of the tumors. On diffusion-weighted imaging (DWI), there was no evidence of restricted diffusion. On magnetic resonance spectroscopy (MRS), all RGNTs showed a slightly elevated choline value and reduced N-acetylaspartate (NAA) value. The mean choline/creatine ratio, NAA/choline ratio, and NAA/creatine ratio were 1.39, 0.61, and 0.45, respectively. No lipid or lactate peak was present. On CT, most of the RGNTs were hypodense (73.3%). The radiological profiles of representative cases are presented in Figures 1–4.

**Table 2:** Radiological features of the RGNTs

	Current study		Literature review		Total	
**Location**	**n**	**%**	**n**	**%**	**n**	**%**
Forth ventricle	10/38	26.3%	62/150	41.3%	72/188	38.3%
Cerebellar vermis	10/38	26.3%	34/150	22.7%	44/188	23.4%
Cerebellar hemisphere	3/38	7.9%	12/150	8.0%	15/188	8.0%
Pineal region/tectum	2/38	5.3%	18/150	12.0%	20/188	10.6%
Third ventricle	1/38	2.6%	15/150	10.0%	16/188	8.5%
Aqueduct	2/38	5.3%	4/150	2.7%	6/188	3.2%
Lateral ventricle	2/38	5.3%	5/150	3.3%	7/188	3.7%
Spinal cord	4/38	10.5%	7/150	4.7%	11/188	5.9%
Thalamus	3/38	7.9%	2/150	1.3%	5/188	2.7%
Suprasellar region	1/38	2.6%	3/150	2.0%	4/188	2.1%
Basal ganglia	1/38	2.6%	1/150	0.7%	2/188	1.1%
CPA	0/38	0%	2/150	1.3%	2/188	1.1%
Brain stem	3/38	7.9%	2/150	1.3%	5/188	2.7%
Septum pellucidum	0/38	0%	2/150	1.3%	2/188	1.1%
Frontal lobe	2/38	5.3%	2/150	1.3%	4/188	2.1%
Temporal lobe	4/38	10.5%	1/150	0.7%	5/188	2.7%
Parietal lobe	0/38	0%	1/150	0.7%	1/188	0.5%
Optic chiasm	0/38	0%	1/150	0.7%	1/188	0.5%
Not mentioned	0	–	3	–	3	–
**Hydrocephalus**	n	%	n	%	n	%
Yes	14/38	36.8%	41/82	50.0%	55/120	45.8%
No	24/38	63.2%	41/82	50.0%	65/120	54.2%
**Satellite lesion(s)**	n	%	n	%	n	%
Yes	1/38	2.6%	7 reported	–	8 reported	–
No	37/38	97.4%	0 reported	–	37 reported	–
**Tumor characteristic on MRI**	n	%	n	%	n	%
Solid	10/38	26.3%	40/103	38.8%	50/141	35.5%
Cystic	9/38	23.7%	16/103	15.5%	25/141	17.7%
Cystic-solid	19/38	50.0%	47/103	45.6%	66/141	46.8%
**Intensity on T1WI**	n	%	n	%	n	%
Hypointensity	36/38	94.7%	69/78	88.5%	105/116	90.5%
Isointensity	0/38	0%	5/78	6.4%	5/116	4.3%
Hyperintensity	0/38	0%	1/78	1.3%	1/116	0.9%
Heterogeneous intensity	2/38	5.3%	3/78	3.8%	5/116	4.3%
**Intensity on T2WI**	n	%	n	%	n	%
Isointensity	0/38	0%	2/74	2.7%	2/112	1.8%
Hyperintensity	33/38	86.8%	61/74	82.4%	94/112	83.9%
Heterogeneous intensity	5/38	13.2%	11/74	14.9%	16/112	14.3%
**Gd-DTPA enhancement**	n	%	n	%	n	%
Non-enhanced	9/38	23.7%	23/91	25.3%	32/129	24.8%
Homogeneous	0/38	0%	5/91	5.5%	5/129	3.9%
Heterogeneous	17/38	44.7%	40/91	43.9%	57/129	44.2%
Rim	9/38	23.7%	11/91	12.1%	20/129	15.5%
Focal	3/38	7.9%	12/91	13.2%	15/129	11.6%
**DWI**	n	%	n	%	n	%
Unlimited	22/22	100%	8/9	88.9%	30/31	96.8%
Limited	0/22	0%	1/9	11.1%	1/31	3.2%
**MRS**	n=12 Range (mean ± SD)		n=1		–	
Choline/creatine ratio	1.04-1.88 (1.39 ± 0.27)		1.3		–	
NAA/creatine ratio	0.42-0.86 (0.61 ± 0.12)		Reduced		–	
NAA/choline ratio	0.32-0.65 (0.45 ± 0.11)		Reduced		–	
**Density on CT**	n	%	n	%	n	%
Hypodensity	11/15	73.3%	18/27	66.7%	29/42	69.0%
Isodensity	0/15	0%	2/27	7.4%	2/42	4.8%
Hyperdensity	0/15	0%	6/27	22.2%	6/42	14.3%
Heterogeneous density	4/15	26.7%	1/27	3.7%	5/42	11.9%
**Calcification on CT**	n	%	n	%	n	%
Yes	1/15	6.7%	8/21	38.1%	9/36	25.0%
No	14/15	93.3%	13/21	61.9%	27/36	75.0%
**Tumor size**	n=38		n=48		n=86	
Range	11-136 mm		5-96 mm		5-136 mm	
Mean ± SD	39.0 mm ± 21.5 mm		32.4 mm ± 17.0 mm		35.3 mm ± 19.3 mm	

### Treatment and immunohistological findings

The detailed treatment approaches, pathological findings, and outcomes are summarized in Table 3. Gross total resection (GTR) and subtotal resection (STR) were achieved in 65.8% and 18.4% of the cases, respectively. In our study, no adjuvant radiotherapy or chemotherapy was administered.

**Table 3:** Treatment, pathology, and prognosis of the RGNTs

Characteristics	Current study		Literature review		Total	
**Treatment**	**n**	**%**	**n**	**%**	**n**	**%**
Biopsy	3/38	7.9%	16/103	15.5%	19/141	13.5%
Partial resection	3/38	7.9%	13/103	12.6%	16/141	11.3%
Subtotal resection	7/38	18.4%	30/103	29.1%	37/141	26.2%
Gross total resection	25/38	65.8%	44/103	42.7%	69/141	48.9%
Chemotherapy	0/38	0%	2/103	1.9%	2/141	1.4%
Radiotherapy	0/38	0%	6/103	5.8%	6/141	4.3%
**Prognosis**	n	%	n	%	n	%
Stable	37/38	97.4%	72/86	83.7%	109/124	87.9%
*In-situ* progression	1/38	2.6%	8/86	9.3%	9/124	7.3%
Dissemination	0/38	0%	2/86	2.3%	2/124	1.6%
Death	0/38	0%	4/86	4.7%	4/124	3.2%
**Follow-up period**	n=38		n=90		n=128	
Range (months)	5-88		2-300		2-300	
Mean ± SD (months)	26.8 ± 19.5		28.5 ± 40.7		28.0 ± 35.7	
**Concomitant pathology**	n	%	n	%	n	%
*In-situ* DNET-like component	5/38	13.2%	6 reported	–	11 reported	–
*In-situ* neurocytoma	0/38	–	1 reported	–	1 reported	–
Astrocytoma	0/38	–	1 reported	–	1 reported	–
Neurofibromatosis type 1	1/38	2.6%	4 reported	–	5 reported	–
Schwannoma	1/38	2.6%	0 reported		1 reported	
Noonan syndrome	0/38	–	1 reported	–	1 reported	–
Multiple sclerosis	0/38	–	1 reported	–	1 reported	–
**Immunostaining markers**	n	%	n	%	n	%
GFAP positive	38/38	100%	117/117	100%	155/155	100%
SYN positive	38/38	100%	116/116	100%	154/154	100%
NeuN positive	13/38	34.2%	8/35	22.9%	21/73	28.8%
Olig-2 positive	38/38	100%	13/13	100%	51/51	100%
MAP-2 positive	38/38	100%	31/32	96.9%	69/70	98.6%
S-100 positive	38/38	100%	35/35	100%	73/73	100%
NSE positive	35/38	92.1%	18/20	90.0%	53/58	91.4%
NF positive	23/38	60.5%	13/38	34.2%	36/76	47.4%
EMA positive	0/38	0%	0/19	0%	0/57	0%
**Genetic variances**	n	%	n	%	n	%
IDH1 positive	0/38	0%	1/18	0%	1/56	1.8%
IDH2 positive	0/38	0%	0/13	0%	0/51	0%
1p/19q deletion	0/38	0%	0/9	0%	0/47	0%
KIAA1549:BRAF fusion	NA	NA	1/17	5.9%	1/17	5.9%
BRAF mutation	NA	NA	0/14	0%	0/14	0%
PIK3CA mutation	NA	NA	6/12	50.0%	6/12	50.0%
FGFR1 mutation	NA	NA	2/6	33.3%	2/6	33.3%
**Proliferation index (Ki-67, %)**	n	%	n	%	n	%
<1%	20/38	52.6%	55/103	53.4%	75/141	53.2%
1–3%	8/38	21.1%	30/103	29.1%	38/141	26.9%
3–5%	7/38	18.4%	11/103	10.7%	18/141	12.8%
>5%^*^	3/38	7.9%	7/103	6.8%	10/141	7.1%

^*^ Ki-67 labeling focally exceeded 5% within rare high-power fields.

*NA*, not available.

**Figure 5:**
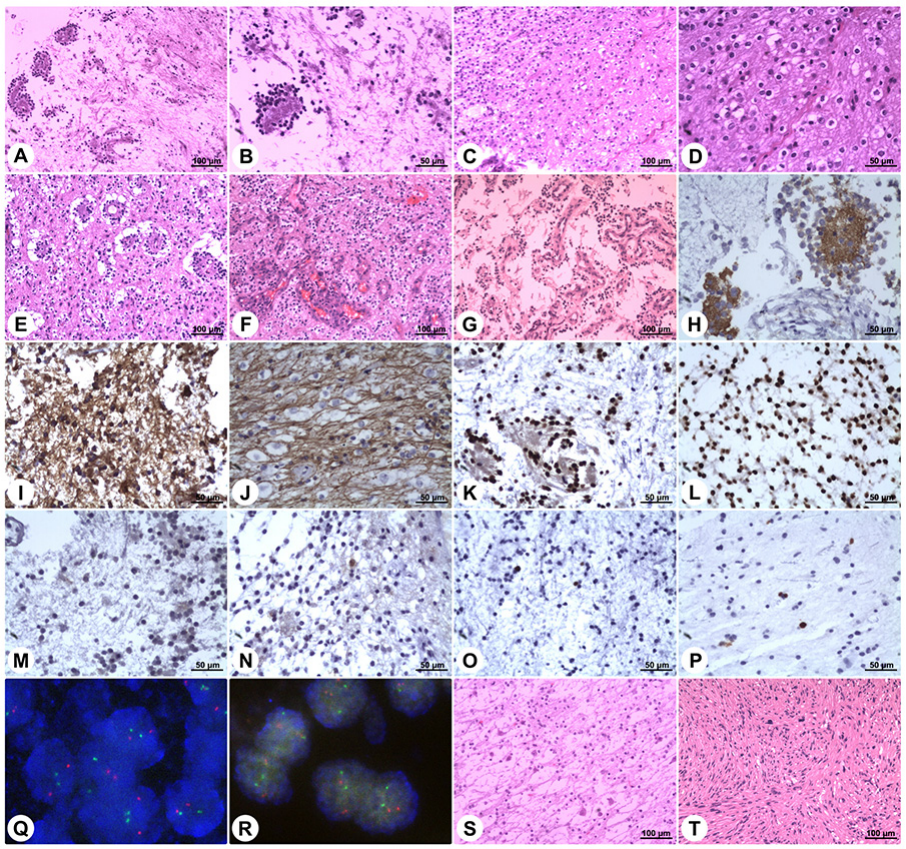
Histopathology and immunohistochemistry of rosette-forming glioneuronal tumors Microphotographs show characteristic histopathological features of RGNT consisting of biphasic glial and neurocytic architecture **(A)**. The neurocytic component is characterized by a ring of tumor cells with scant cytoplasm and dense nuclei, forming rosettes around eosinophilic neuropil cores **(B)**. The glial component consists of spindle- or stellate-shaped astrocytic cells forming a compact fibrillar meshwork with occasional Rosenthal fibers, resembling pilocytic astrocytoma **(C)**. In focal areas of the glial component, oligodendroglial-like cells with round nuclei and perinuclear clear halos are observed **(D)**. Vacuoles are present around the perivascular pseudorosettes **(E)**. Focal microvascular proliferation is observed **(F&G)**. Synaptophysin staining exhibits strong immunoreactivity within the neuropil-like cores of neurocytic rosettes **(H)**. Staining for GFAP demonstrates strong immunoreactivity in the glial background **(I)** and in the oligodendroglial-like component **(J)**. Staining for Olig-2 displays positivity in both the neurocytic rosettes **(K)** and the pilocytic-like glial background **(L)**. NeuN shows focal immunoreactivity in both the neurocytic- and pilocytic-like components **(M&N)**. Ki-67 labeling in both components is low **(O&P)**. Dual-color FISH shows normal disomic status (two red target signals and two green reference signals) of the chromosomes 1p36 **(Q)** and 19q13 **(R)**. In rare cases, the DNET-like component is present, and it consists of the “specific glioneuronal element” with oligodendroglial-like cells arranged in columns separated by microcystic spaces, floating neurons, and mucoid stroma **(S)**. In one case, concomitant spinal schwannoma is found **(T)**. (Stains: A–G = Hematoxylin-eosin stain; H = Synaptophysin immunohistochemistry; I and J = GFAP immunohistochemistry; K and L = Olig-2 immunohistochemistry; M and N = NeuN immunohistochemistry; O and P = Ki-67 immunohistochemistry; Q and R = Dual-color FISH; S and T = Hematoxylin-eosin stain. Original magnification: A, C, E–G, S, and T: 100×; B, D, and H-P: 200×)

Histopathological examination of the specimens disclosed characteristic biphasic neurocytic and glial architectures, leading to the diagnosis of RGNTs (Figure 5). Microscopically, the neurocytic component was composed of uniform small round cells with scant cytoplasm and spherical dense nuclei, and these neurocytes were arrayed surrounding eosinophilic neuropil cores or small vessels forming neurocytic rosettes or perivascular pseudorosettes. The glial element consisted of spindle- or stellate-shaped astrocytic cells with elongated to oval nuclei forming a compact fibrillar meshwork with occasional Rosenthal fibers, and in focal areas, oligodendroglial-like cells with round nuclei and clearly staining cytoplasm were present (Figure 5D), with morphology resembling pilocytic astrocytoma. Cellular atypia, mitotic figures, necrosis, and calcification were rarely visible. Additionally, small foci of glomerulus-like microvascular proliferation were observed in three cases (Figure 5F and 5G).

Immunohistochemical staining showed strong immunoreactivity for synaptophysin (SYN) within the neuropil cores of the neurocytic rosettes and pericapillary neutrophils of the perivascular pseudorosettes (Figure 5H). The glial background, including the astrocytic component and the focal oligodendroglial-like cells, was strongly positive for glial fibrillary acidic protein (GFAP) (Figure 5I and 5J). Both the neurocytic- and pilocytic-like components stained positively for oligodendrocyte transcription factor 2 (Olig-2), S-100 protein, and microtubule-associated protein 2 (MAP-2) (Figure 5K and 5L). Focal immunoreactivity for neuron-specific nuclear protein (NeuN), neuron-specific enolase (NSE), and neurofilament (NF) protein in the neurocytic cells was observed in 13, 35, and 23 cases, respectively (Figure 5M and 5N). Epithelial membrane antigen (EMA) staining was negative in all cases. The proliferation indices according to Ki-67 expression were generally very low ranging from 0% to 5% (Figure 5O and 5P), while focally exceeding 5% (reaching up to a maximum of 8%) within rare high-power fields in three cases. Moreover, no *isocitrate dehydrogenase* (*IDH1/IDH2*) gene mutation was detected, and dual-color fluorescence *in situ* hybridization (FISH) revealed no codeletion of chromosomes 1p36 and 19q13 (Figure 5Q and 5R).

Additionally, we noted *in-situ* DNET-like pathological characteristics in 5 patients, neurofibromatosis type I in one patient, and concomitant solitary spinal schwannoma without neurofibromatosis context in one patient. The DNET-like components consisted of the “specific glioneuronal element” with oligodendroglial-like cells arranged in columns separated by microcystic spaces, floating neurons, and mucoid stroma (Figure 5S). The concomitant spinal schwannoma was found at the C1 level and was resected, while the RGNT in this patient was found in the medulla oblongata (Figure 5T and Figure 4).

### Prognosis

In the current study, remnant tumor progression was noted in one patient 7 months after partial resection, and the other patients were stable at the last follow-up. We searched the relevant literatures and performed a pooled analysis of individual patient data. In the literature, tumor *in-situ* progression/recurrence was reported in nine patients, tumor dissemination was reported in two patients, and a fatal outcome was noted in four patients.

**Figure 6:**
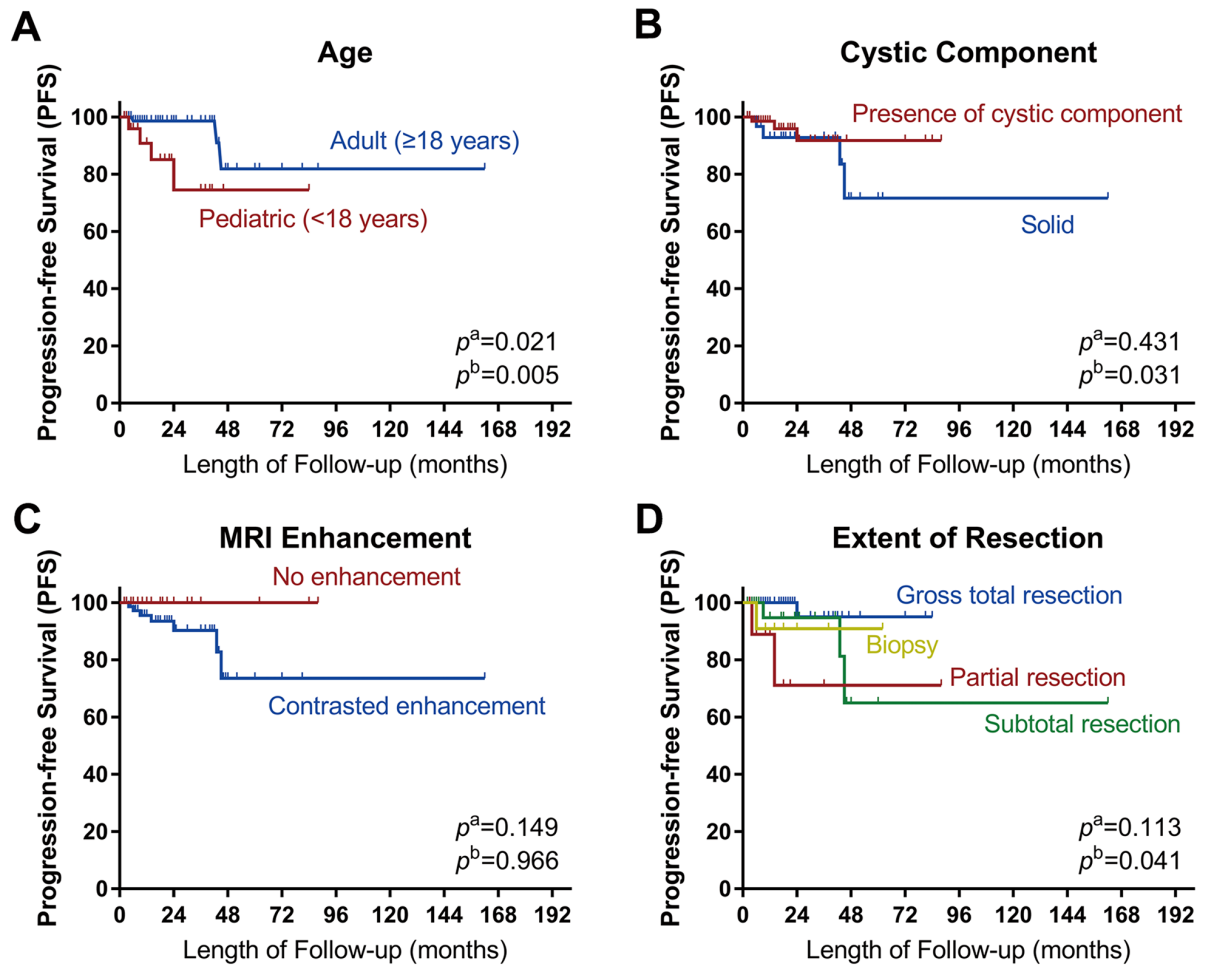
Kaplan–Meier curves Kaplan–Meier curves of progression-free survival for **(A)** age, **(B)** cystic component, **(C)** MRI contrast enhancement, and **(D)** extent of resection. *p*^a^: the *p*-value of Log-rank analysis. *p*^b^: the *p*-value of Cox proportion hazard multivariate analysis. Log-rank tests showed that age was significantly associated with the tumor progression. Multivariate analysis showed that pediatric age, absence of cystic components and inadequate resection extent were significantly associated with the tumor progression.

The statistical results of log-rank tests and Cox proportional hazards analyses for prognostic factors are presented in Table 4. The Kaplan–Meier curves are shown in Figure 6. Log-rank tests and univariate analyses showed that age was significantly associated with progression-free survival (PFS) (HR 0.201, 95% CI 0.044–0.911, *p* = 0.038). Multivariate analysis showed that progression was less likely in adult patients than in pediatric patients (HR 0.003, 95% CI 0.000–0.181, *p* = 0.005). The risk of progression was higher in patients with solid RGNTs than in those with RGNTs having cystic components (HR 78.739, 95% CI 1.479–4192.776, *p* = 0.031). Lastly, the risk of tumor progression was higher with inadequate resection (biopsy or partial resection (PR)) than with GTR (HR 98.258, 95% CI 1.339–7211.531, *p* = 0.036 and HR 155.496, 95% CI 4.336–5575.730, *p* = 0.006, respectively). Patients with STR appeared to do as well as those with GTR (HR 0.655, 95% CI 0.028–15.569, *p* = 0.793). Noteworthily, the Kaplan–Meier curve of PFS for the extent of resection demonstrated mixed results, which was likely due to the limited sample size and follow-up period in this study.

**Table 4:** Log-rank and Cox analysis for PFS*

Variable	PFS				
	Log-rank analysis	Univariate analysis		Multivariate analysis	
	*p*-value	Hazard ratio (95% CI)	*p*-value	Hazard ratio (95% CI)	*p*-value
**Sex**	0.504		0.509		0.071
Male		1^*^		1^*^	
Female		1.659 (0.370-7.444)		7.509 (0.842-67.010)	
**Age**	0.021^†^		0.038^†^		0.005^†^
Pediatric (<18 years)		1^*^		1^*^	
Adult (≥18 years)		0.201 (0.044-0.911)		0.003 (0.000-0.181)	
**Location**	0.447		0.453		0.939
Supratentorial		1^*^		1^*^	
Infratentorial		0.563 (0.126-2.525)		0.902 (0.064-12.650)	
**Cystic component on imaging**	0.431		0.437		0.031^†^
Yes/present		1^*^		1^*^	
No/absent		1.845 (0.394-8.630)		78.739 (1.479-4192.776)	
**MRI contrast enhancement**	0.149		0.376		0.966
Yes/present		1^*^		1^*^	
No/absent		0.033 (0.000-62.395)		0.000 (0.000-)	
**Extent of resection**	0.113		0.229		0.041^†^
GTR		1^*^		1^*^	
STR		5.037 (0.514-49.327)	0.165	0.655 (0.028-15.569)	0.793
PR		12.725 (1.149-140.881)	0.038^†^	155.496 (4.336-5575.730)	0.006^†^
Biopsy		4.755 (0.297-76.107)	0.270	98.258 (1.339-7211.531)	0.036^†^

^*^ “1” designates the reference category.

^†^ Variables significantly associated with the risk of progression (*p* < 0.05).

In the current case series, no patient received adjuvant radiotherapy or chemotherapy. In the literature, four patients underwent postoperative radiation treatment without chemotherapy, of which one died of radiation necrosis and the others were stable during the follow-up period; two patients underwent postoperative combined radio-chemotherapy, of which one gained both clinical and radiological improvement and the other succumbed to tumor progression. The efficacy of postoperative adjuvant treatments could not be estimated owing to the limited sample size.

## DISCUSSION

### Nomenclature and etiology

In 1995, RGNTs were originally described as cerebellar DNTs by Kuchelmeister [15]. In 2002, Komori et al. characterized the clinical, radiological, and histopathological features of RGNTs in 11 cases, and they were the first to propose that these tumors were a distinct clinicopathological entity of mixed glioneuronal tumors [3]. However, thereafter, some scholars reported that RGNTs were not limited to the fourth ventricle and usually presented predominantly or solely with parenchymal involvement [16–19]. Recently, an increasing number of case reports have indicated that RGNTs could also originate from the spinal cord, third ventricle, and supratentorial parenchyma [7, 20–23]. In the current study and literature-based meta-analysis, we found that this entity could occur almost anywhere in the CNS.

The etiology of RGNTs has not yet been clearly elucidated. In previous studies, many scholars speculated that RGNTs might be embryologically derived from the subependymal plate that belongs to the second germinal layer [3, 22]. However, other scholars suggested a potential origin from the cells in the cerebral/cerebellar internal granule layer with the capacity for both neuronal and glial differentiation [8, 18, 24]. Chakraborti et al. proposed a periventricular stem cell origin with biphenotypic differentiation [21]. In the current study, we found that most of the RGNTs were located adjacent to the midline with a few counterparts in the lateral parenchyma, indicating that the above-mentioned hypotheses are tenable, and the definitive pathogenesis requires further embryological research.

### Demography and clinical manifestations

Owing to the extreme rarity of RGNTs, their prevalence in the general population is still unclear. In the literature, RGNTs exhibited a peak incidence in the third decade [4, 25], with a female preponderance (female-to-male ratio, 1.57–1.9:1) [25, 26]. However, in the current comprehensive analysis, the average age at diagnosis was 28.1 years, which is slightly less than that reported previously, and the male-to-female ratio was exactly 1:1, which is inconsistent with the female predominance reported previously [25, 26].

The clinical manifestations of RGNTs are non-specific and localization-related. Corresponding to the main tumor sites of the ventricular system and cerebellar parenchyma, intracranial hypertension (including headache and nausea/vomiting) and cerebellar symptoms are the most common manifestations. When the basal ganglia or the spinal cord is involved, the patients could show sensorimotor disturbance. Of note, hydrocephalus was present in less than half of all cases, and ventricular drain was not performed in most of these cases as there were no severe symptoms of increased intracranial pressure, which may be because of the compensatory mechanism of cerebrospinal fluid circulation associated with the chronic and indolent courses of RGNTs [27].

Interestingly, five cases (including one in the current series) had concomitant neurofibromatosis type I [23, 28–30], and this might not be purely accidental as proposed in a previous study. The definitive association needs further genetic analysis.

### Radiological characteristics

MRI is the preferred examination modality for the preoperative diagnosis of RGNTs [25, 31]. The MR appearance can be divided into cystic, cystic-solid, and solid type, representing 35%, 18%, and 47%, respectively. The cystic components can help in differential diagnosis and may suggest a relatively benign nature, which is supported by statistical results. In most of the RGNT cases, the solid portion showed homogeneous hypointensity on T1WI and homogeneous hyperintensity on T2WI, while contrast enhancement was variable with regard to patterns and degrees of enhancement, and heterogeneous enhancement was the most common. Consistent with the previous literature, on DWI, there was no restricted diffusion uniformly [32]. MRS showed a slightly elevated choline value, reduced NAA value, and absence of lactate or lipid peaks, indicating a low-grade property of RGNTs [31]. On CT, RGNTs showed homogeneous hypodensity or heterogeneous density, and calcification was present in less than a quarter of all cases. Interestingly, a small nodular satellite lesion in the surrounding parenchyma was noted in one of our patients, and satellite lesions have been reported in seven cases in previous studies. A multimodality neuroimaging assessment may help with preoperative differential diagnosis, for example, calcification is more common in oligodendroglioma, and medulloblastoma tends to show hyperdensity on CT and restricted diffusion on DWI [33, 34].

### Histopathological and molecular features

Histologically, RGNTs showed characteristic biphasic neurocytic and glial architectures, and immunohistochemical staining with component-related positivity could help with the diagnosis. The absence of nuclear atypia, mitotic activities, and necrosis, and a low proliferation index in the vast majority of RGNTs indicated a benign biological behavior. However, microvascular proliferation and a high proliferation index in focal areas can be rarely noted, and there is no definitive evidence indicating These features may result in a more aggressive course [10, 35–38]. The molecular features of RGNTs have not yet been well elucidated. Till now, only one case with a *IDH1* mutation [39], one case with *KIAA1549:BRAF* fusion [7], six cases with a *PIK3CA* mutation [24, 40–42], and two cases with an *FGFR1* mutation have been reported [40]. The definite positive rate and distribution of these genetic abnormalities requires further research. Consistently, no *IDH2* mutation or 1p/19q codeletion was detected in both the current analysis and previous studies.

### Therapeutic options and surgical outcomes

Surgical resection remains the mainstay of treatment for RGNTs [10, 23]. However, as most of these tumors are located in midline sites and have an intimate relationship with adjacent key neural structures, especially the cerebellum, brain stem, and spinal cord, it is not always possible to perform complete resection [9, 16, 25, 35]. In the current study, we found that inadequate resection (biopsy or PR) might increase the risk of tumor progression, while there may be no significant difference in progression between GTR and STR. Therefore, we speculate that aggressive surgery with the goal of complete removal, which can have a risk of neurologic injury, may not be necessary.

Although RGNTs are grade I tumors and are considered benign, some reports have presented cases with intraventricular dissemination and rapid progression [4, 12–14, 43]. In the current study, we noted one patient with *in-situ* progression. After systematic review and statistical analyses, we found that pediatric age, purely solid nature of the tumor, and inadequate resection may be risk factors associated with progression events. The efficacies of adjuvant radiotherapy and chemotherapy are yet to be determined for RGNTs owing to limited administration experience [3, 12, 13, 16, 44], and their confirmative role requires further assessment. Although progressive events are rare for RGNTs, clinicians should be aware of these potential events and a long-term close follow-up is needed.

### Limitations of our study

There are several limitations to our study. We collected the individual patient data in our institution and from the published case reports, and performed a pooled analysis using the clinical parameters; however, the inherent heterogeneity and bias (eg. the patients were treated by various surgeons) may influence the statistical power. In addition, several genetic variances (*KIAA1549:BRAF* fusion, *BRAF* mutation, *PIK3CA* mutation and *FGFR1* mutation) have been reported with undetermined significances in sporadic cases, however these variants were not detected in the current study due to financial reasons; this will be our focus in the future research. Another limitation is that the follow-up period is limited, and much longer observation is necessary to make definitive conclusions.

In conclusion, RGNTs can occur nearly anywhere in the CNS, at both supratentorial and infratentorial sites, with a peak incidence in young adults. Certain neuroimaging findings can help preoperative identification. Surgery is the first choice of treatment, and maximal safe surgical resection should be emphasized; meanwhile, aggressive resection with the goal of complete removal may be unnecessary. Pediatric age, a purely solid appearance, and inadequate resection may increase the risk of progression events.

## MATERIALS AND METHODS

### Patients and data collection

This study was approved by the Institutional Review Board and written informed consent was obtained. We enrolled 38 consecutive patients with RGNTs in the central nervous system from August 2009 and June 2016. RGNTs were diagnosed on the basis of pathological criteria, and all slides from the resected specimens, including those used for immunohistochemistry, were reassessed independently by two neuropathologists. The detailed clinical profiles were documented. The MRI and CT characteristics were analyzed independently by two neuroradiologists.

### Treatment

All patients underwent surgical treatment. Based on intraoperative findings and postoperative MR images, GTR (≈ 100% resection by volume) was defined as tumor removal with surgical margins that were grossly and microscopically free of tumor cells. STR (≥ 90% resection by volume) was defined as removal of the majority of the lesion with a small remnant portion in the basal parenchyma. PR (< 90% resection by volume) was defined as removal of regional tumor tissue for decompression. Biopsy was defined as taking a small sample of tumor tissue for histopathological examination.

### Pathological and molecular analyses

Following formalin fixation, paraffin sections of the resected specimens were prepared for hematoxylin and eosin staining and immunohistochemical analysis, including staining for SYN, GFAP, Olig-2, MAP-2, S-100, NeuN, NSE, NF, EMA, and Ki-67. *IDH1/IDH2* gene mutations were examined using immunostaining for mutation-specific antibodies to the R132H substitution of IDH1 and the R172G substitution of IDH2 [45]. Additionally, dual-color FISH analysis was performed on 5-μm-thick tissue sections to analyze the codeletion of 1p36 and 19q13, using previously described methods [14].

### Prognosis assessment

Follow-up data were obtained during individual clinic visits, and the mean follow-up time was 26.8 months (SD = 19.5; range, 5–88 months). Follow-up MRI scans were requested at 3 months after surgery, semi-annually for 2 years, and annually or every 2 years thereafter. As the sample size of the present study was limited, we analyzed the influence of various factors on prognosis after synthesizing the current data and individual patient data on published cases in the literature.

### Pooled analysis of individual patient data and statistics

We searched PubMed, Embase, and Web of Science (up to and including December 2016) for published articles using the search terms “rosette AND forming AND glioneuronal” and identified 146 papers. The search yielded 108 papers reporting a total of 153 RGNT cases. Individual patient information was collected and analyzed.

Qualitative data are described as counts and percentages, and quantitative data are described as mean (standard deviation, range). PFS was defined as the period from treatment to evidence of tumor progression on imaging or tumor-related death. Patients who were event-free at the last follow-up and those who died from causes unrelated to tumor progression or relapse were considered as a censored event. PFS was evaluated using the Kaplan–Meier estimator, and results were compared using the log-rank test. A Cox proportional hazards model was fitted to identify the prognostic factors of PFS. Hazard ratios with corresponding 95% confidence intervals were calculated. Univariate and multivariate analyses were performed by inserting variables, including sex, age (pediatric or adult), tumor location (supratentorial or infratentorial), cystic component (present or absent), MRI contrast enhancement (present or absent), and extent of resection (biopsy, PR, STR, or GTR), in order to explore the influence of these factors on PFS. The patients with incomplete follow-up data were excluded from the Kaplan–Meier and Cox model analyses. All statistical analyses were performed using SPSS software (version 24.0; IBM Corp., Armonk, NY). P-values of <0.05 were considered significant.

## Abbreviations

RGNT: rosette-forming glioneuronal tumor
T1WI: T1-weighted imaging
T2WI: T2-weighted imaging
DWI: diffusion-weighted imaging
MRS: magnetic resonance spectroscopy
NAA: N-acetylaspartate
GTR: gross total resection
SYN: synaptophysin
GFAP: glial fibrillary acidic protein
Olig-2: oligodendrocyte transcription factor 2
MAP-2: microtubule-associated protein 2
NeuN: neuron-specific nuclear protein
NSE: neuron-specific enolase
NF: neurofilament
EMA: epithelial membrane antigen
IDH1/IDH2: isocitrate dehydrogenase
FISH: fluorescence *in situ* hybridization
DNET: dysembryoplastic neuroepithelial tumor
PFS: progression-free survival
NF1: neurofibromatosis type I
ADC: apparent diffusion coefficient
